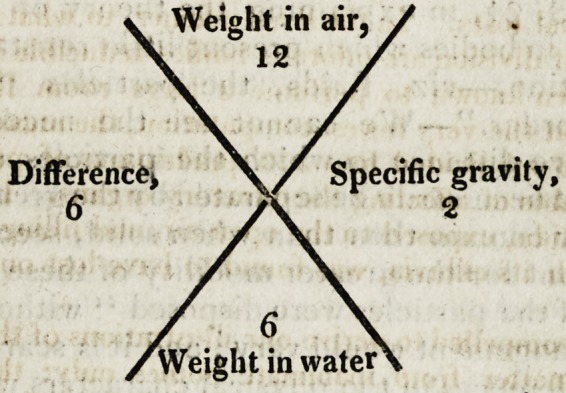# Book Reviews

**Published:** 1825-07

**Authors:** 


### CRITICAL ANALYSIS
### OF
### ENGLISH AND FOREIGN LITERATURE,
### RELATIVE TO THE VARIOUS BRANCHES OF
### Medical Science.

Quæ laudanda forent, et quæ culpanda, vicissim

Illa, prius, cretâ; mox hæc, carbone, *notamus.*—PERSIUS.

---

### DIVISION I.
### ENGLISH.

ART. I.—*The Elements of Medical Chemistry; embracing only those Branches of Chemical Science which are calculated to illustrate or explain the different Objects of Medicine; and to furnish a Chemical Grammar to the Author's Pharmacologia. Illustrated by numerous Engravings on Wood.* By JOHN AYRTON PARIS, M.D. F.R.S. F.L.S. Fellow of the Royal College of Physicians of London; Honorary Member of the Board of Agriculture; Fellow of the Philosophical Society of Cambridge; and of the Royal Medical Society of Edinburgh; and late Senior Physician to the Westminster Hospital. —8vo. pp. 586. W. Phillips, London, 1825.

No branch of natural science has made such rapid progress during the last half century as chemistry. So rapid, indeed, has been, and still continues, its advancement, that it requires considerable exertion, even for those conversant with the subject, to acquire a knowledge of the new facts which the labours of the experimentalist are daily bringing to light. Let any chemist slumber over his studies for a single year, and the probability is, he will find himself as much behind as, in any other science, he would be in half a century. Contemplating, too, the almost boundless field for investigation, the immense number of bodies with which the chemist is supposed to be familiar; the multifarious relations they bear to each other; and the endless modifications of which they are susceptible, in different forms of combination;—we say, considering all this, it must be acknowledged that there is no branch of science which the medical pupil is called upon to study, in which his opportunities and means of acquiring it are so little commensurate to the end,—and, consequently, we believe there is none in which medical men are, in general, so deficient. The difficulty in acquiring a competent knowledge of chemistry consists not in any thing abstruse or perplexing in its general principles, which are, indeed, easily apprehended by any ordinary mind; but, as stated above, it consists in the almost innumerable forms of matter with which it is necessary to become acquainted. Another unfortunate circumstance resulting from the extensive nature of the subject, is the impossibility of doing justice to it in any course of Lectures, of the length prescribed by usage, and generally adopted in this country: the student is hurried on to the history of individual bodies before he has acquired any competent knowledge of general principles; and, toiling in vain to follow the rapid course of the teacher, who must get over the ground within a given time, he becomes discouraged and disgusted. We would earnestly recommend the division of these courses into one purely elementary, and one for those more advanced.

The work before us is the only one we have in this country, which professes to confine its details to those branches of the subject "which are calculated to illustrate or explain the different objects of medicine;" and, as we think it eminently calculated to facilitate the acquisition of chemical knowledge among our brethren, we are desirous to give it whatever benefit it may derive from being brought under the notice of our readers, with the humble mead of our earnest commendation.

Before entering upon the more immediate business of the volume, Dr. Paris shows, in an introductory chapter, the extensive nature of the application of chemical to medical science.

"The science of chemistry may be said to be subservient to medicine, in demonstrating the various changes which occur in the component parts of the animal body, under the different conditions of health and disease; and in appreciating and explaining the phenomena accompanying such changes;—in investigating the composition and occasional deterioration of the air we breathe, and of the various solid and fluid substances which we employ as aliment or medicine;—in suggesting processes of art by which natural bodies may be adapted, or new and artificial compounds produced, for administration as remedies;—in detecting the presence, and counteracting the effects, of various noxious substances which may, either from accident or design, become instrumental in impairing health, or in destroying life;—and, lastly, in instructing the practitioner how he may best direct the admixture and combination of various remedies, without the risk of occasioning such changes in their properties as may alter or invalidate their efficacy." (P. 4, 5.)

Attempts have frequently been made to impress chemistry into the service both of physiology and pathology; and the numerous absurdities into which this has led are too well known to require illustration. The result has probably been to drive us somewhat too far into the opposite extreme, and to deny to the chemical agencies of bodies their due share in the phenomena of living functions.

"Nor must it be forgotten that, in some of the functions of the living body, the vital energy would seem rather to correspond in its action with chemical affinity, than to oppose or supersede its influence; and several of the senses may be said to owe their energies to the perfection of organs which are entirely constructed upon philosophical principles. Thus are the laws of optics and acoustics in active operation during the exercise of the visual and auditory apparatus; and it is a question whether some chemical action is not established by the agency of sapid bodies upon the epidermis of the mucous membrane of the mouth: it is, at least, seen evidently in some cases, as in the effects of vinegar, the mineral acids, a great number of salts, &c. By the same agents, similar effects are produced upon dead bodies; and Dr. Magendie thinks that to this species of combination the different kinds of impression made by sapid bodies may be fairly attributed, as well as the variable duration of such impressions. Nor is it reasonable to deny that many of our remedies may act by a chemical action on the alimentary canal: alkalies are thus frequently serviceable, by clearing the primæ viæ of superfluous animal matter, which they effect by forming with it a soluble compound. If the origin of animal heat cannot be satisfactorily traced to a strictly chemical source, its maintenance, distribution, and regulation, may, at least, be shown to depend upon the agency of those laws which alike govern the temperature of inert matter. Do we not perceive that every animal, suffering from diminished temperature, instinctively diminishes the surface of its body, which is in contact with the cooling medium? Man, under such circumstances, is seen to bend the different parts of his limbs upon each other, and to apply them forcibly to the trunk. It will be also seen, when we come to consider the nature of capillary action, that many of the phenomena of living bodies, which have been erroneously attributed to the action of the living principle, may be satisfactorily explained by the simple operation of this attractive force. The absurdities of the chemical and mechanical sects have undoubtedly driven the modern physiologist into a mischievous scepticism with regard to the influence of physical causes upon a living animal: John Hunter even (to associate whose name with error will be regarded by many as an act little short of impiety,) has repeatedly attributed to the specific effect of *life*, actions that ought to be solely referred to the powers belonging to inanimate matter. In the same manner, an objection to impute to the physical property of elasticity certain phenomena exhibited by membranous structure, has led Bordieu, Bichat, Blumenbach, and others, to assign to it a peculiar vital power, whose existence has neither been proved by experiment, nor rendered probable by analogy." (P. 7—9.)

To the pathologist, likewise, the doctrines of chemistry will frequently be found capable of affording important assistance; and, as the illustrations of these applications are both interesting and ingenious, we shall make no apology for laying them before our readers.

"But there is another point of view in which the same question may be advantageously regarded: the pathologist will have to contemplate the living powers in various states of languor and decay, when they will be found incapable of wholly resisting the laws which govern inanimate matter; and we shall learn, during the progress of the present work, that, in certain conditions of the human body, several of the fluids will undergo the same chemical decompositions as would take place in the laboratory. The same observation will apply to the agency of mechanical causes. In a state of perfect health, the fluids of the body will not descend to the inferior parts, agreeably to the law of gravitation, because the vital power opposes itself to this hydraulic phenomenon, and with an energy in direct proportion, as it would seem, to the robust and vigorous state of the individual; for, if the person be reduced by disease, this tendency will be only imperfectly resisted: the feet, in consequence will swell. The following experiment of Richerand may be here related, to show how greatly the power manifested in the living body of resisting, with more or less success, the influence of physical force, is enfeebled by disease. He applied bags filled with very hot sand all along the leg and foot of a man who had just undergone the operation for popliteal aneurism: the artery was tied in two places under the ham. Not only was the usual coldness which follows an interruption of the circulation thus prevented, but the extremity so managed acquired a degree of heat much greater than the ordinary temperature of the body. The same apparatus, when applied to a healthy limb, was unable to produce that excess of caloric, obviously in consequence of the energy of life opposing such an effect.

Mr. Earle has published an interesting paper to prove that, when a limb is deprived of its due share of vitality, it is incapable of supporting any fixed temperature, and is peculiarly liable to partake of the heat of surrounding media. The cases which are adduced prove also that a member so circumstanced cannot, without material injury, sustain a degree of heat, which would be perfectly harmless, or even agreeable, to a healthy part: thus, the arm of a person became paralytic, in consequence of an injury of the axillary plexus of nerves, from a fracture of the collar-bone; upon keeping the limb for nearly half an hour in a tub of warm grains, 'which were previously ascertained by the other hand not to be too hot,' the whole hand became blistered in a most alarming manner, and sloughs formed at the extremities of the fingers. In a second case, the ulnar nerve had been divided by the surgeon, for the cure of a painful affection of the arm; the consequence of which operation was, that the patient was incapable of washing in water at a temperature that was quite harmless to every duly vitalised part, without suffering from vesication and sloughs." (P. 9—11.)

To the pharmacologist, the necessity of chemistry is so obvious, that we shall not dwell upon it; and, in concluding this part of the subject, we would only mention one illustration which is given of the confirmation which the observations made at the bedside of the patient, occasionally derive from the experiments made in the laboratory. Most of our readers will remember the interest which the Eau Medicinale excited some years ago, and the numerous attempts made to detect its composition. It was conjectured by Mr. JAMES MOORE that hellebore was the active ingredient in this nostrum; and the trials made at the Westminster Hospital induced our author to regard this opinion as correct. Soon after, the colchicum autumnale was discovered to be its basis, and some degree of ridicule was attached to those who had regarded the hellebore as possessing analogous properties. How completely is this charge of careless observation removed by the recent and singular discovery, that both the hellebore and meadow saffron contain the same active ingredient—*veratria.*

The plan adopted in the work before us is very simple: the general principles are first explained, and next we are made acquainted with the properties of individual bodies. As the principles of medical chemistry differ in nothing from those of the science in general, so, under the former department, we have those elementary details common to all similar works. The subject, however, is not treated of in a manner exactly similar to that adopted in any work with which we are acquainted. We have more of natural philosophy than is usually admitted in works *ex professo* on chemistry; a circumstance which renders the details more useful and more interesting: we have, for example, some curious and interesting illustrations of the divisibility of matter, better calculated to satisfy the mind of the student than any thing in form of abstract demonstration.

"Divisibility is a property which belongs to every substance which can be brought under the cognisance of our senses, but it by no means follows that matter in its elementary state possesses it: indeed, it is more probable that at some term, however distant, the resulting particles lapse into simple atoms incapable of any further resolution. If marble, or any brittle substance, be reduced to the most impalpable powder which art can produce, its original particles will not be bruised or affected; since, if this powder be examined by a microscope, each grain will be found a solid stone, similar in appearance to the block from whence it was broken; and, of course, if we possessed suitable implements, would admit of being again subdivided, or reduced to a still finer powder. To what extent this reduction might be carried before we arrived at the simple elementary atom, we shall probably never be able to conjecture; for the divisibility of matter, if not infinite, at least exceeds the utmost limits of our imagination. The marble steps of the great churches in Italy are worn by the incessant crawling of abject devotees: nay, the hands and feet of bronze statues are, in the lapse of ages, wasted away by the ardent kisses of innumerable pilgrims that resort to those shrines. What an evanescent pellicle of the metal, says Mr. Leslie, must be abraded at each successive contact! Thus, again, a single grain of the sulphate of copper will communicate a fine azure tint to five gallons of water; in which case the copper must be, at least, attenuated ten million times, and yet each drop of the liquid may contain as many coloured particles, distinguishable by our unassisted vision; and, if the experiment be extended by still further dilution, so that the metal shall cease to be an object of sense, it may nevertheless be recognised by chemical tests. In the same manner, to what a most extraordinary degree of division are odorous bodies reducible? A single grain of musk has been known to perfume a large room for the space of twenty years: at the very lowest computation, the musk, in such a case, must have been subdivided into 320 quadrillions of particles, each of which was capable of affecting the olfactory organs. In like manner, a lump of assafœtida, exposed to the open air, and filling the surrounding atmosphere with its effluvia, was found to have lost only a single grain in seven years.

Nor are we compelled to derive our illustrations of the almost infinite divisibility of matter from inanimate bodies only: the naturalist and physiologist will supply us with a multitude of striking examples from the vegetable and animal kingdoms. How extremely minute, for instance, must be the parts of the seed by which the peculiar plant constituting mouldiness is propagated? for Reaumur found this production in the interior of an addled egg, whence the seeds must have passed through the pores of the shell! Mr. Leewenhoeck has informed us, that there are more animals in the putrifying milt of a cod-fish, than there are men on the whole earth, and that a single grain of sand is larger than four millions of these creatures, so that thousands of them could be lifted on the point of a needle, and yet each individual must be provided with a series of organs: of what inconceivable minuteness, then, must be the ultimate fibres of such organs! But the infusory animalcules, as they are termed, display, in their structure and functions, the most transcendent attenuation of matter. The Vibrio undula, found in duck-weed, is computed to be ten thousand million times smaller than a hemp-seed. The Vibrio lincola occurs in vegetable infusions, every drop of which contains myriads of these points. The Monas gelatinosa, discovered in ditch-water, appears, in the field of a microscope, a mere atom endued with life, millions of which are seen playing, like the sun beams, in a single drop of liquid.

"The human structure likewise affords many wonderful instances of similar attenuation. The red globules of the blood have an irregular roundish shape, from the 2500th to the 3300th of an inch in diameter, with a dark spot in the centre of each. The globules of perspirable matter have been computed as, at least, ten times smaller than those of the blood, each being about the 5000th part of an inch in diameter; and, when the quantity of perspirable matter which is daily discharged, and the number of pores through which it passes, are estimated, it will follow that no fewer than 400 of such globules must issue from each orifice every second." (P. 16–20.)

Under the head of Gravitation, we have a very full and satisfactory account of the various methods of taking the specific gravities of bodies; an operation which, if more generally practised, would be found of great use to the pharmaceutical chemist, particularly in detecting frauds and adulteration. By a very simple method, the calculation is considerably facilitated: it consists merely in registering the steps of the process in the alternate angles of a cross. Thus,—suppose a piece of sulphur to be weighed in air, and its weight to be 12 grains; the amount lost in water is next ascertained,—say 6. The real weight 12, divided by the loss 6, of course gives the specific gravity 2. This plan of registering the results is convenient, and the whole account of the different methods of ascertaining specific gravities, as well as the other processes described in the course of the work, are illustrated by numerous diagrams and wood-cuts. We would likewise point out the history of the Atomic Theory as particularly good: it is usually very perplexing to the student, but is here explained so well, that he cannot fail to comprehend it very readily.

It is, of course, impossible to give any comprehensive account of an elementary volume of this kind, and we must content ourselves with pointing out some of the circumstances in which it differs from other works of a similar kind.

It is, then, in the second part, that the student will find his interest and convenience most attended to: here all that does not relate to medicine is thrown aside, by which the memory is released from an immense, and to many an intolerable, burden; and he will find the history of all those bodies he is called upon to know, following each other in succession, and not scattered about in various parts, as is the case in every general system of Chemistry.

Having spoken thus favourably of the work, we shall now mark in succession some of the passages which have appeared to us less satisfactory, in the hope that, in a second edition, the objections, if well founded, may be obviated.

At page 16, it is said that, "besides the properties above enumerated, matter is said to possess certain secondary, or contingent, properties, such as elasticity, fluidity, &c. and which, by their combination with the general properties, constitute the condition or state of bodies."—Quere, can properties be said to combine with each other? One property may be superadded to another; many may be co-existent; but we apprehend nothing which, in strict language, can be called combination ever takes place.

At pages 23, 24, in explaining the theory of Polarity, it is supposed that in bodies which present little resistance to motion in any direction,—viz. fluids, the particles "are disposed without any order."—We cannot see the necessity for this: the comparative distance to which the particles of bodies in a fluid state must necessarily be separated by the greater proportion of caloric they contain then than when solid, seems to us sufficient to account for the greater mobility of these particles upon each other. If the particles were disposed "without any order," then their arrangement would vary; and it is scarcely to be supposed that differences in the physical characters would not result from some of these variations. If the particular form of disorder did not vary, but was always the same, then we could no longer say that they were disposed "without any order," but that the want of order was only apparent, and that fluids, like solids, are governed by the laws of polarity, and "always exhibit the same arrangement and disposition of parts."

At page 211, speaking of the heat evolved by friction, it is said, "nor is it owing to combustion of oxygen, with the bodies themselves, or with any part of them."—*Combustion* we apprehend to be a typographical error, and that it ought to be *combination.*

At page 268, we are informed that, "during *every* combustion in oxygen gas, the gas suffers a considerable diminution in volume;" and this, it is further stated, may be illustrated by burning phosphorus, or "*any inflammable body,*" in a jar of oxygen gas.—Now this, if stated as a general proposition, would be correct, but it is not so when made universal. During the combustion of charcoal in oxygen, no diminution in the volume of the gas takes place; and, if the student attempted to verify the proposition by this experiment, he would probably be rather perplexed by the result.

At page 394, it is said of lime, that, when pure, "its colour is *grey;*" and the general whiteness in the colour of lime is imputed to its absorbing moisture and a portion of carbonic acid.—We very much question the accuracy of this, at least as regards the colour of pure lime, which we believe to be *white;* although it be true that *common* quick lime be comes somewhat whiter on the addition of water.

At page 396, we find the following paragraph:—

"*Metals of the third class*.—These metals, of which arsenic and antimony alone possess any interest to the medical student, are distinguished from all others in the property which they possess of becoming acidified by a certain degree of oxidation. With other proportions, however, of oxygen, some of these metals assume the ordinary characters of *oxides*. This is remarkably exemplified in the history of antimony, which forms with oxygen two very distinct orders of compounds. In the first, they act the part of a salifiable base, as illustrated by tartar emetic; in the second, that of an acid, neutralising the acids and other bases, and giving origin to the salts called *antimonites* and *antimoniates*."

—We do not understand the last sentence of this quotation, and apprehend that there is some mistake in it. What is the meaning of a body, which acts the part of an acid, "*neutralising the acids and other bases?*" One acid can never be said to neutralise another; and, with respect to the antimonious and antimonic acids here spoken of, the former combines with acids sparingly, and the latter appears to be incapable of combining with them at all.

At page 445, of carbonate of lime it is said, that, "when subjected to a strong heat, the carbonic acid is driven off, and the *base* of lime remains;" whereas, lime itself remains. It is probably a typographical error, and ought to be rectified.

These, however, are trivial circumstances, which interfere little, if at all, with the general character of the work: a more serious objection, as it appears to us, is the arrangement. Of this the reader may form an idea, from the following account of it:—

"The following is an outline of the arrangement which I have usually followed in my Lectures, in treating of the different simple and compound substances of nature and art. Water is presented as the first object of examination, not only as being a fluid universally known, but as entering very generally into the composition of other bodies, and as influencing, by its presence, all the phenomena of chemistry. Its decomposition, moreover, brings the student at once acquainted with two leading elements, oxygen and hydrogen,—the nature of which, from the important part which they perform, cannot be too early understood; while their gaseous form will afford us an opportunity of illustrating the methods to be adopted in collecting, transferring, and examining gases. Carbon, which may be said to constitute the basis of organised matter, is conidered next, and, by its union with oxygen and hydrogen, we shall learn the nature and properties of carbonic oxide, carbonic acid, and carburetted hydrogen. In this part of our arrangement, the consideration of the Atmosphere may be entertained, since, with the exception of azote, it includes no principles whose nature has not been already explained. Under this head the theories of Combustion, of Animal Heat, and of Oxygenation, may be conveniently introduced. The history of the simple supporters of combustion, Chlorine and Iodine, and their combinations, will follow. The compounds produced by the union of Oxygen and Azote, of Hydrogen and Azote, and of Carbon and Azote, will succeed. The histories of Sulphur, Phosphorus, the Metals, and Neutral Salts, will conclude the first part. The second and third divisions will comprise the subject of Organic Chemistry." (P. 248.)

Now, the disadvantages attending this, and almost every other arrangement adopted by modern chemists, is, that bodies of the most opposite properties are brought together, while those of similar character are separated. Every one, for example, has been accustomed to hear of alkalies and earths, on the one hand, and acids, on the other; and, to what extent soever the discoveries of chemistry may be carried, these will remain as two great classes of bodies, the individuals of which we naturally associate together in our minds, as possessed of certain common properties; and, when we come to study their history scientifically, we expect to find them following each other in succession, preceded by an account of their general characters. It is this adoption of the natural associations which gives, in our judgment, a great superiority to the arrangement of the late Dr. Murray, —at least to the student. We are quite aware that the other, and more modern, plans of Thomson, Henry, Brande, and our author, may be justified on the plea of greater scientific accuracy. There appears, however, to be something very arbitrary in placing ammonia between the hypo-nitrous and hydrocyanic acids, as in the work before us; nor do we like to see our old acquaintances, potass and soda, in the secondary rank of the oxides of the metals, although this is not peculiar to Dr. Paris. With regard to the salts, finding them arranged according to their bases, we turned to the article "Salts of Lead," and were rather startled to find no mention made of the acetate. On searching, however, under the head "Acetic Acid," we found it along with the other acetates. Now, to give a consistent view of the subject, the salts ought to be arranged, either according to their bases, or according to the acid which forms them; but, to distribute them partly in the one way, and partly in the other, gives rise to some degree of confusion. In a science which undergoes such rapid changes, it is in vain that absolute accuracy is sought after in any arrangement, and therefore we ought to adopt that which most facilitates the study. For this purpose, we have never met with any which appeared to us so good as that adopted by the late Dr. Murray.

"There are three works," says Dr. Paris, "which every student, desirous of becoming an accomplished chemist, should possess: Henry's Elements of Chemistry, Brande's Manual, and Dr. Ure's Chemical Dictionary." Without undervaluing any of these, we may be allowed to say that, to the pupil who is beginning to study, we would recommend Mrs. Marcet's "Conversations on Chemistry;" next let him study the "Elements of Medical Chemistry," by Dr. Paris; and, if he chooses to prosecute the subject further, he may consult any of the three above mentioned, or (what certainly is inferior to none of them) Thomson's "System of Chemistry."

We conclude in the words of our author, sincerely hoping that the expectations held out may be fulfilled at no distant period: —"The student might now proceed to the consideration of Animal Chemistry, but, as the present volume has already exceeded the limits assigned to it, and as the extent of this branch of science would require several hundred pages for its investigation, I have determined to defer it until a more favourable opportunity will enable me to treat of the characters and composition of animal products, and to show the changes which the fluids of the body undergo during the influence of disease."

---

ART. II.—*Observations on the Use of the Colchicum Autumnale in the Treatment of Gout; and on the proper Means of preventing the Recurrence of that Disorder.* By CHARLES SCUDAMORE, M.D. F.R.S. Member of the College of Physicians in London; Honorary Member of Trinity College, Dublin; of the Medico-Chirurgical Society of Edinburgh; and of the Medical Society of Paris; Member of the Medico-Chirurgical Society of London; Physician in ordinary to his Royal Highness the Prince Leopold of Saxe Coburg, &c. &c. —8vo. pp. 116. Longman and Co. London, 1825.

ALTHOUGH the observations of Dr. SCUDAMORE may, from the title-page, be expected to be limited to the prevention of gout, and the use of the meadow-saffron in that disease, yet the subjects treated of, and the points of theory and practice discussed, in this little volume, are so very various, that they pass before us, like the views of the *peristrephic panorama*, in fleeting succession, without possessing the due connexion which would convince us that they belong to the professed subject of discussion, or the "interesting medical question" proposed. There is neither an index nor table of contents, but a laconic heading at the top of each page denotes the matter treated of in the text below, in the following order:—"Use of Colchicum; Theory of Gout; Doctrine of the Ancients; Practice of the Ancients; Greek Manuscript; Hermodactyl of the Ancients; Sydenham on Gout; Kinglake on Gout; Eau Medicinale; Colchicum

Autumnale; Wilson's Tincture; Reynolds' Specific; Empirical Treatment; Chemical Analysis; Wilson's Tincture; Reynolds' Specific; Preparations of Colchicum; Experimental Inquiry; Pathological Principles; Pathology of Gout; Empirical Treatment; Regular Treatment; Pathological Principles and Case; on Sarsaparilla; Sulphate of Quinine; Error of Digestive Functions; Principles of Treatment; Use of Mercury; Use of Aperients; Obscurity of Bilious Complaints; Principles of Treatment; Preventive Treatment; Principles of Diet; Use of Wine; Preventive Use of Medicine; Treatment of the Paroxysm; Local Treatment; Conclusion."

From this enumeration of the contents of the volume before us, in the order they are treated of, it is evident that the author digresses from, and returns to, any subject of his observation, without paying attention to any regular rule of composition, or that established succession of topics usually observed by writers who consider method and order conducive to the illustration of their observations, and to their more ready comprehension, and to a greater facility of retention in the memory. This eccentricity of movement bids defiance to a regular analysis, and the want of the usual classical arrangement prevents us from following our author step by step in the devious path he has laid out. We shall, therefore, endeavour to bring into one view the author's sentiments on any particular point under review, however scattered those sentiments may be in the volume before us.

Having stated that an indiscriminate employment of colchicum by most gouty persons, must have led to frequent mischief, and that vague rumours prevail of evil consequences resulting from its influence, Dr. S. unfolds his object in the following paragraph:—

"I shall endeavour to treat the subject in a manner quite intelligible to the general reader; for, although I wish also to make it a medical investigation, and to address myself to the medical public, yet it is so obvious that gouty patients take upon themselves the treatment of their own cases, that, in my opinion, they stand in need of a clear exposition of the true bearings of the question from a medical pen." (P. 2, 3.)

As the papers printed in different Numbers of this Journal have not only been the principal means of reviving the use of colchicum autumnale in gout, but have pointed out safe and mild modes of its administration, we may be supposed to feel considerable interest on this important subject, and will be excused from indulging a bold and free criticism on any observations that may be made on it.

"Previously to my observations," says Dr. S. "on the merits of colchicum, and upon the treatment of gout, I shall offer some remarks on the nature of the disease, in order that my principles of practice may be clearly understood.

"We cannot too steadily keep in view, that the peculiar external appearance of complaint which we denominate gout, is the least part of the disease, and is to be regarded as the symptom of some error in the constitution. The local suffering is the effect of a cause which is existing in the system. This fundamental position being admitted, our next inquiry is, what is the nature of the constitutional cause which gives rise to this specific inflammation in the extremities? The knowledge of the proximate cause, or that one essential and indispensable state of the system which is the invariable antecedent of the disease, is an interesting problem, which baffles our most acute research." (P. 3, 4.)

At pages 7 and 49, this theoretical doctrine is repeated,—"The gout, even in its first visitation, is a compound disease, external in its appearance, internal in its cause."

When Dr. S. admonishes us to "constantly keep in view, that all which appears externally, and to which the name of gout is given, is but the least part of the real disease," if we did not disregard his admonition, and dissent from the doctrine, we are quite satisfied that "the general reader" will, when labouring under a severe gouty inflammation of the joints of the extremities, (the excruciating pain of which has so frequently occupied the imagination of medical writers, to discover comparisons sufficiently strong to convey a just idea of its tortures.) When an extremity or joint is enormously swollen, inflamed, and painful, or the skin is in a state bordering upon mortification, it would be very difficult to make either the practitioner or patient subscribe to the creed which inculcates these external appearances, or local complaints, to be the least part of the disease. If it should so happen that this local disease, by the application of "Dr. Kinglake's cold water," or any other cause, be transferred to the stomach, lungs, heart, or head, would any one be induced to believe the local malady to be the least part of the disease; or would any one so tenaciously adhere to the doctrine, that "the local suffering is the effect of a cause which is existing in the system," as to direct his practical attention to the removal of this constitutional cause, instead of vigorously attacking and relieving the local suffering and symptoms. This theory is, in fact, refuted by the context; for we are told in the same paragraph, "that the knowledge of this cause is an interesting problem, which baffles our most acute research." Surely, no philosophical reasoner is entitled to concede a powerful agency to a visionary cause, which eludes the most acute research; nor to give it form and substance, by embodying it into the shape of a gastro-hepatic pathology, as is subsequently attempted. Indeed, we are told "we must rest satisfied with the fact that some individuals possess an hereditary disposition to gout, and others acquire this disposition wholly by means of improper habits of living acting upon that

*peculiarity of constitution* which involves the inscrutable question of proximate cause! Nevertheless, agreeably to our author's experience, many, whose habits of life are marked by irregularity and excess, escape the gout.

The theory of gout is closed by a paragraph, purporting to be a reply to an interesting question, but which we cannot perceive contains any answer at all.

"In answer to the positive and very interesting question, what is gout? I shall, with the premises already advanced, avoid the discussion of proximate cause, and direct my view to all the most obvious, most characteristic, and most clearly marked, circumstances which distinguish the disease." (P. 6.)

The first circumstance noticed is, that "the paroxysm often takes the patient by surprise, and finds him in the enjoyment of health, or (more correctly speaking) possessing comfortable feelings, and those good looks which give the portrait of health; although, most assuredly, the constitution is not really in a healthy state." (P. 7.)

Dr. S. next "proceeds to observe, that the kind of constitutional error which attends a first fit of gout, is an overcharged state of the vessels which belong to the abdominal viscera, and chiefly in the vessels connected with the liver; the secreting action of this important organ being also, in some way, altered from its usual state. The stomach itself is the organ least deviating from its ordinary and healthy state. The appetite very commonly continues natural; unless, indeed, the symptoms are sufficiently severe to produce high sympathetic fever, when thirst and loss of appetite would naturally follow. The *stomach*, it is true, has been the *parent source of the gout*, in being the medium through which too much animal material has been introduced into the system; but it seldom suffers itself any sensible inconvenience in the first instance." (P. 8.)

Dyspepsia is eventually produced; too much abdominal fulness is induced; the veins of the extremities are unnaturally distended; a want of due softness and pliancy in the right hypochondrium, (where the general reader is told the principal part of the liver is situated!) may be discovered upon close investigation; the muscles are much covered with fat; the secretions of the digestive organs, and of the kidneys, become morbidly changed, so that "we derive a conclusion that the process of assimilation is not perfectly performed, and that the blood itself is not in a natural and healthy condition." (P. 10, 11.)

That there are some gouty patients to whom Dr. S.'s description will apply, cannot be denied; but our experience authorises us to affirm, that there are many in whom the above circumstances and symptoms are not "the most obvious, most characteristic, and most clearly marked; for gout supervenes in numerous spare habits, whose muscles are not clothed with fat, who are not corpulent, and whose modes of living are not only necessarily temperate, but exemplarily abstemious; as may be instanced among females and spare-fed paupers, in whose system "the parent source of gout, the stomach," does not introduce too much animal material, and in whom there is not the slightest evidence of "fulness of vessels."

Dr. S. principally insists that gout, even in its simplest form, is connected with "that species of repletion which belongs to the vessels of the abdominal viscera, and chiefly of the liver; and that, in a very large proportion of cases, the symptoms of gout are entirely supported by great derangement of the biliary system, and by an unhealthy condition of the intestinal canal. We coincide so far with Dr. S. in the belief that, in chronic cases of long duration, the functions of the liver are, or become, more or less unhealthy; but we may affirm, with equal truth, that the hepatic and digestive functions become deranged in every case of chronic disease belonging to the order Phlegmasiæ, attended with pain; and there appear to be stronger reasons for believing that the local symptoms of gout, if protracted, produce and prolong hepatic derangement, than that disorder of the biliary functions causes gout. Because hundreds and thousands of cases of biliary and chylopoietic derangement are met with in practice, in which gout does not ensue; whilst the generality of cases of gout, accompanied with active local inflammation, are attended with loss of appetite, furred tongue, and unhealthy secretions of the bowels,—as in rheumatism and other instances of inflammatory disease. Besides, many cases of gout occur, in which none, or very little, chylopoietic disturbance supervenes, in consequence of the mildness of the local symptoms.

Our author next takes a brief review of the doctrines and practice of the ancients. Of the moderns, he only mentions Dr. Kinglake, whose practice of applying cold water to gouty parts he deters us from following, by the appalling statement, that "this practice, for a considerable time, had its bold advocates; but, having made some fatal victims, and often given warnings of danger, in producing sudden sensations of internal spasm, it has been universally laid aside; and scarcely any one can now be found who does not, as regards this particular treatment, give way to the dictates of prudence and common sense." (P. 19, 20.)

We at length arrive at Dr. S.'s observations on the colchicum autumnale and its varied preparations, whether in the form of powder, tincture, spirit, wine, or extract, or designated by the name of eau medicinale, Wilson's tincture, Want's remedy, or Reynolds' specific.

It is, at once, assumed that the colchicum autumnale is the hermodactyl of the ancients: Sir J. Banks was of the same opinion, and, as he obtained the hermodactyl from Syria, he was probably correct; for, in Salmon's Dispensatory of 1682, "the true eastern hermodactyl, with a dry white root, is called also colchicum, being pleasant and sweet: it purgeth tough phlegm from the joints, and is *a specific in the gout of hands or feet.*" In a Pharmacopœia of the London College of Physicians, belonging to the Medical Society of London, edited in 1625, the hermodactylus and colchium autumnale are enumerated as distinct bulbs, or roots; and, in a manuscript note, the colchicum autumnale is styled the pseudo hermodactylus. Indeed, medical writers did not agree on the subject; for MATHIOLUS and others thought the iris, or orris-root, the true hermodactyl; whilst LOBEL. DODON and others denied it. Although the eastern hermodactyl, or colchicum autumnale, had obtained a reputation, in centuries past, for the cure of gout, in the various forms of electuary, powder, of aqueous and acetous infusion, cataplasm, &c., and this *anima articulorum* formed the principal ingredient in the Vinum antipodagrieum Mynsichti, in the blessed laxative of Nicolai, the Theriaca arthriditis Avicennæ, the Pulv. arthriticus Turneri, and the Dec. arthrit. Viennæ, yet it had fallen into disuse, probably from the exhibition of such large and injudicious doses as produced violent and injurious effects on the stomach and bowels: for we find, in Salmon's Dispensatory, that the dose advised was from ten to thirty grains of the powder, and from two to four drachms of the infusion.

The bulb of the colchicum autumnale was not only considered a specific for carrying off the gout, but some preparations of it are recommended to be repeated every month for keeping it off: such are the Pulv. arthrit. Turneri, the Vin. arthrit., the Cerevisia arthrit., and the Pulv. hermodactylus Paracelsi.

Mr. WANT, who was formerly editor of this Journal, published some papers in it between 1811 and 1814, from which it appears he investigated the opinions of the ancient writers, and their practice in the treatment of gout; and, chiefly from the recommendation of P. ÆGINETA and ALEXANDER of Tralles, he was led to make trial of the colchicum autumnale, which proved successful, and terminated in the revival of the use of that remedy. Its employment, however, was slowly and cautiously introduced into practice, and very gradually adopted by the profession at large; notwithstanding its efficacy was experimentally proved, by many professional and other eminent men of the present age, on their own persons. Apprehensions were entertained that the exact doses and precise quantity of the medicine necessary were not ascertained, and that its deleterious and inflammatory effects on the stomach and bowels might prove injurious or fatal.

Mr. Bampfield, in two papers published in the 46th and 47th volumes of this Journal, proved, by several cases, that very small doses of the colchicum autumnale,—as, for instance, twenty or thirty minims of the wine, given once a-day for three or four successive days, were adequate to the gradual cure of the gouty paroxysm, without producing any other sensible effect than the gradual subsidence of all gout, pain, and inflammation, or any other gouty symptoms that might be present. Hence it was inferred that such small doses might be administered with safety, with a mildness of operation that quieted the alarms of the most timid, and with a certainty of success that would not disappoint the hopes of the physician or his patient. As Dr. S. entered into a short controversy with Mr. Bampfield on the subject of his paper, it is presumed he must be aware of the colchicum wine having been exhibited in such small doses, and of its having thus carried off the paroxysm in a slow and gradual manner. We were, therefore, surprised to find "the principle of practice" sneered at as "empirical," when "any of these powerful agents be used,—namely, the eau medicinale, Wilson's tincture, Reynolds' specific, or the strong preparations of colchicum. These remedies are almost always employed in a manner to procure their full and immediate influence on the nervous system; an effect which may be called their most specific mode of acting; the sole object being to arrest at once the progress of the disease, and dismiss the symptoms, without any inquiry as to the particular state of constitution in the individual." (P. 27.)

It is clear that this statement is at variance with what has been previously observed, and, we would say, with the practice of most of those who employ it.

To return from this historical digression to our author. "It has been a very natural question," says he, "asked on all sides, what is the composition of the different nostrums, and in what essential particular do they differ?" As Dr. S. includes the "strong preparations of colchicum" in the list of nostrums;" and, as the College of Physicians have recently admitted two of them, the vin. colchici and sp. colch. ammon., into their legitimate list of formulas, and have, moreover, adopted the recipe of Sir E. Home for manufacturing the vin. colchici, against which our author has thundered his anathema over and over again, the College will no doubt vindicate itself, and their opinion in favour of the vin. colchici cannot fail of having due weight with the profession, notwithstanding it is branded as a nostrum.

"From my experimental inquiry, I assume the conclusion, that the eau medicinale, Wilson's tincture, and Reynolds' specific, are all preparations of colchicum of different degrees of strength; and further, that the tincture of colchicum, the wine of the roots as commonly prepared, the wine of the seeds, and the vinous preparation directed by Sir Everard Home, must be enlisted with the three nostrums, if employed in a manner to produce their most distinct and specific agency." (P. 52.)

As no satisfactory analysis of vegetable tinctures or wines can be accomplished, it is impossible thus to identify the different preparations possessing a specific effect in gout. Dr. S., therefore, draws his inference of their identity (as Mr. Want and all others have done, whose writings precede this volume,) "from the influence they exert on the human constitution;" or, in more precise language, from the power of all to relieve, and carry off, the gouty paroxysm, with a certainty that no other known medicine can be said to possess. The author is, therefore, eventually "led to the essential conclusion, that the eau medicinale, Wilson's tincture, and Reynolds' specific, are all preparations of colchicum; and that the compositions of hellebore and opium, and of elaterium and opium, are not identical with the eau medicinale.

The remarks on the frequent injurious effects of some of the nostrums, including the known preparations of colchicum, are passed over; as we are satisfied the injurious result is attributable, in the use of all, to their exhibition in doses unsuitably large, or at an improper stage of the disorder; for small doses seldom produce any sensible effect whatever, in gout or any other disease.

From the third edition of Dr. S.'s "Treatise on Gout and Rheumatism," he has here republished an account of the effects on the dog of all the preparations of colchicum, as well as of elaterium and hellebore with opium. "Most of these medicines (as most of our readers already know,) were injected into the jugular vein, and were also administered by the mouth," and produced "high irritation of the nervous system; the pulse and breathing much disturbed; sickness; a discharge from the bowels of blood and mucus; inflammation of the stomach and intestinal canal; and death." (P. 38.) The acetum colchici, however, proved to be comparatively gentle and mild in its effects, as well as the extract prepared from it. These experiments appear to prove also that the sediment of the vinous infusion of colchicum is completely inert; which Sir E. Home was induced, from his experiments, to conclude, possessed highly acrid and poisonous qualities.

From the whole tenor of our author's observations, the medical reader will naturally expect to hear that Dr. S. continues to give the decided preference to his gout-draught, of which the acetum colchici forms an essential ingredient, notwithstanding the recent introduction of the two new preparations of colchicum into the London Pharmacopœia, which is a strong counter-expression of the sense of the College in their favour. Some instances are adduced of the digestive functions remaining disordered after the gouty paroxysm has been hastily carried off, and others where relapses sooner followed from this practice; and we agree with him in stating, that " it is highly important that the cure should be gradually conducted."

The effects of producing a habit of relapse by an improper use of large doses of the legitimate or empirical preparations of colchicum, are said to be the following:—

" The nervous system has been much shaken, the patient being depressed in mind and body; the limbs have been weakened and tender; the muscles thin and relaxed; the digestive functions impaired; the bowels in a most unhealthy state; the action of the kidneys morbid and irregular.

" This is a faint, rather than a strong, picture of the unhappy results of the improper treatment of gout." (P. 56, 57.)

For the information of the general reader, (it is presumed,) the author observes,—

" When the constitution has been brought into this serious and confirmed state of error, I have found it extremely difficult to conquer the tendency to gout, and to restore the disordered functions to health; but in every instance (and such cases have been very numerous,) in which the patient has given me his complete confidence, and persevered with the means to the full extent of my wishes, I have reaped for him, and for myself, the reward of complete success." (P. 57.)

Some cases are related, with a view of showing the superiority of what is termed the *regular* treatment, as a contrast to that denominated *empirical* treatment; in which the main difference appears to consist in carrying off the paroxysm more gradually, and, should any derangement of visceral function continue afterwards, suitable remedies are to be prescribed,—such as aperients, alterative doses of mercury, sarsaparilla, and tonics. But the author shall speak for himself.

" The cases of gout which I have here briefly stated, have been those examples of troublesome relapse which have required a particular method of treatment, including, in the list of remedies, the use of the acetic preparation of colchicum, administered occasionally in conjunction with correctives and aperients, during the prevalence of gouty inflammation." (P. 66.)

The propriety of preventing a fit of gout is next considered.

"In my Treatise, I have laid it down as a principle, that we should attempt the prevention of a fit of gout, if warned of its approach; and interrupt its progress when formed, unless a state of constitution exist, implying that the gout has taken the place of another more serious disease, or may be expected to prevent one which is threatening, and more to be dreaded than itself: but, even in this event of gout, it is highly proper to moderate the violence of symptoms, and protect the system from excess of pain and irritation.

"With regard to the question of soliciting the gout, in order that it may take the place of another disease, we are to consider whether it is proper to use any stimulating means, in reference to the existing disease. For example, if a gouty patient have an inflammation of the lungs, bleeding and the usual means of treatment are to be practised, with a freedom corresponding with the force of the symptoms; and, if any means were used to invite the gout, they should be local only,—as the use of a pediluvium with hot water, flour of mustard, and bay-salt; and of liniment consisting of the linim. camph. comp. et tinct. lyttæ." (P. 67, 68.)

A case of suppressed gout follows, in which our author was eventually led to prescribe, as a tonic, the infusion of the cortical part of sarsaparilla in lime-water, and afterwards the sulphate of quinine in a suitable vehicle; from which circumstance permission is claimed "to digress a little on the merits of sarsaparilla, and of the sulphate of quinine, as medicines of restorative and tonic power." (P. 77.) From which it appears, the experience of our author induces him "to entertain a very favourable opinion of the medicinal qualities of sarsaparilla," and that he has prescribed the sulphate of quinine "with very great success, as a tonic for the gouty patient, in the stage of convalescence.

"The following is a formula which I have most commonly used. It is agreeable to the palate, and almost invariably suits the stomach.

℞ Sulphat. Quinin. gr. jss.

Infus. Rosæ, ℥x.

Sp. Myrist.

Syr. Aurant. āā ʒj.

Acidi Sulph. dilut. gr. ij. M. Ft. haustus.

For every additional grain of the sulphate of quinine, it is useful to add two drops of the diluted sulphuric acid, which answers the purpose sufficiently of keeping the salt in solution. I do not consider that the useful properties of the quinine become impaired by the slight combination which it forms with the astringent principle of the rose." (P. 83, 84.)

The error of the digestive functions of gouty persons receives a brief notice, in the treatment of which nothing novel presents itself; from which subject our author passes on, and "is tempted to offer some remarks on the use of mercury.'

"I consider it an axiom of importance, that mercurial medicines should never be administered to gouty persons, to any extent which carries with it the risk of producing mercurial fever." (P. 89.)

We cannot, however, perceive how this risk is to be avoided, if patients be treated like the one at page 74, in which "repeated doses of calomel, James's powder, and compound extract of colocynth, were administered at bed-time."

After a few observations "on the use of aperients," and "the use of mercury in obscure bilious complaints," in which there is nothing that is not generally understood, "the prophylaxis, or the care which can be most usefully taken to prevent the recurrence of gout, by the general management of the constitution," comes under discussion. It must be familiar to most of us, that the gout is frequently the offspring of dietetic indulgence and irregularity; whilst, on the other hand, the utmost care and prudence, and the regularity even of youth, sometimes prove insufficient to prevent the first attack of gout, or its returns. Simplicity of diet is recommended to those who desire to counteract the returns of the disorder. The diet should not be too nutritious, or disposed to acetous fermentation. "In *(from)* principle, also, many articles of food should be avoided by the gouty patient;" of which are mentioned salmon and stewed fish, pork, hard and salted meats, pickles, salads, rich pastry, most confectionary, rich soups, and oysters. The gouty patient is also advised to "confine himself to the very careful use of good stomachic wine, matured by time,—as sherry, madeira, or port, a little diluted with water." (P. 105.)

"The preventive use of medicine" is limited to the employment of some gentle aperient, the best suited to the constitution of each patient.

"I would lay it down as a general rule, that every gouty person, however regular the state of his bowels may be, should take some aperient at least once a-week throughout the year, and, if he be corpulent and of full habit, twice a-week; and further that, in every instance, it is important that the bowels act once a-day, as a constant rule; and those who are corpulent, or much prone to gout (unless debilitated), should study to produce the habit of action night and morning." (P. 108.)

Our author again reverts to the treatment of a severe paroxysm of gout, by observing, "I need not repeat my objection to the employment of any of the various nostrums, or the strong preparations of colchicum. I have also explained the grounds on which I recommend the use of the acetum colchici, neutralized and joined with an aperient salt." (P. 109.) The dose of the acetum colchici is from half a drachm to one and a half. Hence we perceive that Dr. S. continues a faithful adherent to his gout-draught and the acetum colchici, to the utter exclusion of all other preparations of meadow-saffron, except the acetous extract; notwithstanding the College of Physicians has sanctioned the employment of two of the strong preparations of colchicum, and that the professional world is universally satisfied of their safety, mildness, and efficacy, in small and well-regulated doses.

The work concludes with a recommendation of the evaporating lotion noticed in his former Treatise, and of the ordinary remedies employed for the removal of stiffness and weakness of the limbs,—such as baths, liniments, friction, and shampooing.

From the numerous extracts we have made, and the account given of the contents of this volume, our readers will be convinced that it contains no sentiments, and but little matter, that have not appeared in the author's Treatise on Gout, if we except the history of three or four cases of gout; the brief, and rather irrelevant, notice of the sulphate of quinine; and the less doubtful, although qualified, admission of the specific agency of colchicum in gout. It seems, therefore, to be intended for the general rather than the medical reader; an opinion in which we became the more confirmed by the language quoted from page 57 and other passages, and from the author having thought it necessary to give a trite description of the vena portæ and its office.

---

### DIVISION II.

### FOREIGN.

Art. III.—*A Compendious System of Midwifery, chiefly designed to facilitate the Inquiries of those who may be pursuing this Branch of Study. Illustrated by occasional Cases; with fourteen Engravings.* By W. P. Dewees, m.d. Lecturer on Midwifery, Member of the American Phil. Soc. &c.—8vo. pp. 628. Miller, London, 1825.

We now enter upon the consideration of a subject which we consider of the very highest importance, and of which a perfect knowledge can only be acquired by deliberate study and considerable practice. The physician who has not particularly devoted his attention to the science of midwifery, may enter boldly into *discussions* respecting the management of difficult labours, and may even go so far as to detail, with respectable accuracy, the manner in which manual assistance is to be afforded. To talk, is an easy task. A man may possess a perfect abstract knowledge of a subject, without being enabled to bring it into action when suddenly called upon. The merest tyro in the profession, with but a very moderate share of application, may learn, in a very short time, all the steps of a difficult operation. But bring his knowledge to the test. Is it probable—is it possible, that he can at once possess the adroitness of an experienced surgeon. It may appear a mere waste of time to argue such a question for a single moment. We have heard it, however, contended,—and publicly contended, too,—that no practical experience is necessary in the practice of midwifery; that the general physician, provided he be duly instructed in the fundamental principles of his profession, is as fully adequate to undertake the management of difficult labours, as he who has directed his undivided attention to the subject. The manual assistance, however, which an accoucheur is called upon to offer in complicated and preternatural cases, requires as much dexterity, and infinitely more patient exertion of mind, than the general operations of surgery. It might be ungracious to inquire into the motives which can induce particular individuals, or collective bodies, to degrade, by every means in their power, the practitioners of midwifery. HIPPOCRATES is not less renowned, because he exerted his splendid ability in the practice of obstetrics; nor (to come nearer to our own times,) was the fame of HARVEY sullied by exercising this branch of medicine. How is the importance of any part of our professional duties to be fairly estimated? The answer is incontrovertible. We are useful to the public, and we reflect honour upon ourselves in proportion to the suffering we alleviate, and the number we rescue from impending danger. We leave entirely out of the question the interest we feel, as men, in the alleviation of the distresses of women in child-bed. No class of practitioners can be more useful to suffering humanity, than those who dedicate their attention to midwifery; and it is as ridiculous as it is mischievous to assert, that the duties of their art can be properly performed, without much of their time being exclusively devoted to the subject.

Dr. DEWEES has been long known in this country, although he is separated from us by the Atlantic Ocean: the very first volume of our Journal contains a valuable contribution from his pen. It is natural that we should approach the work of such a veteran in his art with respect. He has given frequent proofs of the ability he possesses, and of the laudable zeal which animates him. Although we labour in the service of the *indocti* as well as the *docti*, we do not feel called upon to enter upon the detailed consideration of the introductory matters with which every general Treatise must commence. We look upon it as a duty, however, to peruse them with attention, that we may not sanction, by our silence, errors which would mislead the student who might rely with confidence upon that which had not been pointed out as unworthy of it. We shall touch but lightly, then, upon the preliminary chapter, which describes the *structure of the pelvis;* wishing it, at the same time, to be deeply impressed upon the mind of every obstetrical student, that he cannot practise with advantage to his patient, nor with gratification to himself, without a perfect knowledge of all the parts which are interested in the important process of labour. The pelvis, it is well known, is subject to many diseases at different periods of life, which alter its natural measurement, and which present obstacles, of more or less importance, to the birth of the child.

"But," says Dr. Dewees, "as every degree of deviation does not render labour impracticable by the natural agents of delivery at full time, it will be well to set the boundary which the practitioners of Europe, of the greatest experience, have affixed for it; and it seems to be pretty generally conceded, that a labour cannot, successfully to the child, be effected, when there is less than three inches in the antero-posterior diameter of the superior strait. When a pelvis has three inches, or even three inches and a half, in this diameter, the labour is rendered for the most part tedious, painful, and uncertain. We hear of some remarkable cases, however, of children being born alive, when there has been but two inches and three-quarters from the pubes to the sacrum; but these must constantly be regarded as exceptions to the general rule, and require, that it may take place, an unusual suppleness in the bones of the cranium." (P. 18, 19.)

Our author has appealed to the "practitioners of Europe," because crooked women are not to be found in America, and, therefore, the "united experience of all the American practitioners would not have led to a correct conclusion upon the subject." He states, that whenever deformity has occurred to "such extent as to render labour impracticable by the natural powers, it has uniformly been with European women. He is not singular in this opinion. We wish he had endeavoured to explain the reason why American women particularly should be comparatively exempted from such serious afflictions. Does it arise from any difference of education, or treatment during the early periods of their lives, when the foundation is laid, in most instances, for pelvic deformity: and, if it does, in what do these differences consist? Dr. Denman was of opinion, that, when deformity of the pelvis exists, it is invariably in the small diameter of the superior aperture, and never in the direction of the great one. Dr. Dewees, however, has met with two instances of this kind in his own practice, and "is in possession of a natural pelvis, where the diameters of the upper strait are reversed." Such cases are very rare.

Tumors and exostoses within the pelvis occasionally give rise to difficult, or even impracticable labour. Such cases are not of frequent occurrence, but, as the life of the patient will depend upon the judgment of the practitioner when they do occur, and probably upon the promptitude with which his knowledge may be brought into action, it is incumbent upon us to be intimately acquainted with the best practical rules upon the subject. Dr. Dewees has had but little experience upon it. Mr. Burns may be consulted with much advantage, as may Capuron.*

It is frequently of considerable importance that we should be enabled to ascertain the extent of the different diameters of the pelvis. To effect so useful an object, many instruments have been contrived,—such as the pelvimeter of Contouli, the caliper, upon which Baudelocque places so much reliance, &c. &c. It is not possible, however, with any of these instruments, to arrive at a correct admeasurement of the pelvis; and we would answer the observation of our author, that "we may, with very considerable accuracy, determine the antero-posterior diameter, by the introduction of the finger into the vagina, and placing its extremity against the most projecting part of the base of the sacrum, and allowing the radial arch of it to press against the arch of the pubes," that more than once, in cases of pelvic deformity, in which several eminent accoucheurs were called in, sufficient proof of the uncertainty even of this mode of admeasurement has been afforded us. Each practitioner has formed a different opinion of the dimensions. Upon this subject we must also observe, that we have not only to determine where, and to what extent, pelvic deformity exists; the difficulty or impracticability of the case will depend also upon the size of the head of the child, and upon the state of the bones of the cranium, which can only be ascertained at an advanced period of labour. It will be well for the young practitioner to bear in mind, during his management of a natural case, that the hour of difficulty will sooner or later arrive. If he be extensively occupied, he will, of course, have his share of obstacles to surmount, and perplexities to contend with. Let him, then, take advantage of the opportunity which every labour affords him of exercising his practical tact, and of determining in his own mind the dimensions of the pelvis, and he will, with comparative facility, although not perhaps with positive certainty, determine the nature and extent of the variation from the natural structure of the part.

The difficulty and duration of labour will frequently depend upon the position of the head within the pelvis. This is not to be determined without a habit of deliberate examination during labour, and an accurate knowledge of the parts. To know that the head presents is not sufficient, although, from repeated facts which have come within our own observation, we are convinced that a great majority of practitioners are satisfied if

* *Traité d'Accouchemens,* p. 630. 1811.

they can but feel any part of the cranium. The author truly remarks, that

"No man can with any certainty render assistance, where the head has departed from its proper route, who shall be incapable of distinguishing by the touch this aberration: he will either not distinguish the faulty position, and thus condemn the poor woman to protracted and unnecessary suffering, or he will blindly and rashly attempt relief, to the hazard of the lives of mother and child.

"Many rely upon the position of the ear, for the knowledge of the situation of the head; but we very loudly object to this test:—1st. Because it may be so high in the pelvis, as to be out of the reach of the finger; 2d, it may be so impacted in the pelvis, as to prevent the finger from passing to it; 3d, that, when felt, it may give, from some peculiarity of situation, a wrong impression of its position; 4th, that, when the head is still enclosed within the uterus, the finger cannot always be made to pass under the edge of it sufficiently far to reach it, though the os uteri is sufficiently dilated for all the purposes of delivery.

"It is important that the connexion of the head with the trunk should also be well understood; otherwise much injury, if not death, may be incurred from an ignorance of it: it must be constantly recollected that the head cannot with safety execute a motion beyond a quarter of a circle, when it is freed from the pelvis, and the body retained within that cavity; nor can the cervical vertebræ more safely perform a greater sweep, when the head is detained, and the body without. A want of attention to this fact, we have great reason to fear, has caused the death of more children than we would dare to mention, especially when they have presented by the breech, feet, or knees. We well recollect one instance of footling presentation when the child was delivered to the head: the midwife, who had the charge of the case, could not succeed in delivering this; we were sent for, and we were obliged to give two entire turns of the body before the twist was removed from the neck. We need not mention the fate of the child. There are fewer errors commited of this kind when the head presents; not because the cases are not equal under equal circumstances, but because the shoulders are seldom long retained after the exit of the head, and consequently there is less temptation to employ ill-directed force." (P. 30—31.)

*Of the Uterus and its dependencies.*—We will enter no further into the often-disputed question of the structure of the uterus, than by offering our assent to the opinion of Dr. Dewees, that "sufficient is known of its structure to warrant the declaration that its functions, as regards labour, are performed by the power of muscular contraction. The following suggestions as to the independence of different parts of the uterus upon each other, are ingenious, and peculiar to our author.

"The division of the uterus into different portions, was suggested for the convenience of demonstration, and has been employed by all the writers upon either anatomy or midwifery, for at least the last century:

we adhere to this division, but from very different motives than the one just alluded to. Many years ago we insisted on this division as essential to the explanation of several of the phenomena which this organ constantly presents: we shall therefore transcribe, without apology, our sentiments, as expressed upon this subject, in an 'Essay on the Means of lessening Pain, and facilitating certain Cases of difficult Labour,' p. 17, ed. 2d.

"'I cannot help regarding the neck of the uterus as a distinct and independent part from the body and fundus, as having its own peculiar laws and actions; and that this separation of powers is absolutely necessary to the explanation of some of the phenomena exhibited in health and in disease, and the influence of certain agents upon this organ.

"'My reasons for thinking so are, first, that we find the fundus and body may be distended to a great extent, without affecting the arrangement of the neck: thus, in every uterine pregnancy, we see these parts gradually yield to the influence of the ovum, until about the sixth or seventh month; while the neck remains very much the same as before impregnation.

"'Secondly, that, after the sixth or seventh month, the neck undergoes its changes, while the fundus and body remain in a great measure stationary; so that two distinct processes, or rather the same process, is performed at two different periods, and in different parts, in the order we have just mentioned.

"'Thirdly, that the neck may be affected by disease, while the fundus and body may remain free, and the reverse; and that the neck may contract and relax, while the other parts are in opposite states. Thus, with women who are in the habit of aborting, from some peculiarity of the uterus, we find the body and fundus may be excited to action, while the neck for a long while remains passive; and also the neck may relax, and after some time the fundus and body may be excited to contraction. And, in cases of atony of the uterus after a too sudden delivery, the body and fundus may contract, while the neck is the only part in fault, and *vice versâ.*

"'The different conditions that the parts of the uterus may be in at the same time, where atony partially prevails, would seem to demonstrate the truth of what is here advanced. For it is a fact well known to almost every practitioner of midwifery, that each of the parts into which we have divided this viscus may, separately and independently of each other, be in a state of relaxation or contraction, and thus exhibit different phenomena, and be productive of different results.' From this it would appear that nature has really established a division of the uterus, which has hitherto been considered as merely conventional." (P. 38–40.)

We must pass over the consideration of the various hypotheses with respect to the nature and causes of the *menstrual flux.* A cursory sketch is given of the different opinions which have successively "lived to die" upon this interesting, but obscure, subject. We have had extensive opportunities of noticing the derangements arising from irregular menstruation, but have not met with cases precisely similar to those referred to by our author.

"We have," he says, "known several instances, where the eruption of the menses were constantly preceded by strong hysterical paroxysms, of greater or less permanency: the menses would now appear, and instantly the system would be tranquillised, and the woman return to her ordinary state of health. One case we knew where a severe pruritus accompanied this convulsive state, to the great annoyance of the poor young creature who was the subject of it." (P. 46.)

In a note we are informed that "this young woman was perfectly relieved from these disagreeable symptoms, by the use of camphor at the commencement of the menstruous period, and liberally washing the parts, in the interval, with a strong solution of borax."

The "use of camphor" is a very unsatisfactory phrase. We believe the powers of this medicine to be infinitely under-rated, from the doses in which it is usually given being much too small. For some information upon this subject, we refer to a paper of Mr. RING's, in the sixth volume of our Journal, page 156. Mr. Ring thinks highly of the remedy in cases of painful menstruation. He administered it in ten-grain doses. It is usually considered sufficient to give the mist. camphoræ of the Pharmacopœia, which has more taste, but no more virtue in such cases than so much water. In such instances as those mentioned by our author, we can recommend camphor from our own experience. We should be unwilling to give ten grains at a dose, without having first tried, by the exhibition of a smaller quantity, the power of the patient to bear the remedy. We cannot promise invariable success from it.

In the chapter *on Conception*, Dr. Dewees repeats the conjecture he promulgated many years ago. He believes "in the direct conveyance of the semen, by being taken up from the labia pudendi or vagina by a set of vessels, whose whole duty is to convey it to the ovaries." We may add, that this idea is in part confirmed by the discovery of ducts leading from the ovaria to the vagina in the cow and sow, by Dr. GARTNER, of Copenhagen.

In commenting upon the different kinds of *action of the uterus* which that organ is capable of exerting, Dr. Dewees states it as his opinion that the ordinary contraction of the uterus, which is brought into play for the purpose of expelling its contents, is attended by pain, "not as an inevitable consequence of this contraction, but that it is owing to some change the muscular fibre has undergone, from civilisation, refinement, or disease. We readily admit that we comply with the artificial, and frequently absurd, customs of civilised society, which are too imperative to be easily escaped from, with the loss of much of that original vigour of body which characterised our forefathers, and with the *gain* of many diseases, of which they were perfectly unacquainted. We agree that labour is rendered a more painful process by departing from the simple ordinances of nature, with respect to the management of the female during childhood,—her subsequent education,—and, lastly, her style of dress. But we have yet to learn upon what authority the opinion rests, that labour was ever borne without more or less suffering being felt from the throes which necessarily attend it. Neither do we consider the statement accurate, "that in the brute this contraction is successfully excited without the intervention of pain, unless the labour be complicated with disease or accident." As far as we can judge from the motions of a parturient animal,—from the expression of its countenance, which is as indicative of suffering as the human,—the creature does suffer, although it would be difficult to estimate the degree of pain nature has inflicted during the fulfilment of this all-important process.

Such cases as the following are not uncommon.

"In 1792," says the author, "we were called to attend a Mrs. C., in consequence of her midwife being engaged. As we approached the house, we were most earnestly solicited to hasten in, as not a moment was to be lost. We were suddenly shown into Mrs. C.'s chamber, and our appearance explained by stating that her midwife was engaged. As we entered the room, Mrs. C. was just recovering from a pain; and it was the last she had at that time. After waiting an hour in the expectation of a return of labour, we took our leave, and were not again summoned to her for precisely two weeks. Every accoucheur has experienced the temporary suspension of pain upon his first appearance in the sick chamber, but for the period of two weeks is very rare." (P. 74.)

In no department of practice is it so necessary to steer clear of a positive opinion, as with respect to the duration of a labour, whatever may be the progress that has been made, short of the expulsion of the head. In several instances we have known the confidence of a family lost, from an assurance being given that labour would be terminated at a stated time, when, from the sudden and unexpected cessation of uterine action, the patient has gone on for several days. Experience will doubtless enable us to form a tolerably correct opinion in the majority of instances, and we may cheer the patient with the hopes of being relieved from her suffering; but we should at all times provide a retreat for ourselves by not hazarding to name a particular time,—unless, indeed, (to use the words of a late able teacher,) "we have the business in our hands, because we have fairly hold of the head."

*Retroversion of the Uterus.*—This species of displacement of the uterus is not regarded in the same view by different practitioners: "Hunter, Baudelocque, Meygrier, Burns, &c. look upon it as an accident of serious moment; others, as Denman and Merriman, view it almost with careless indifference. As both cannot be right, we shall, in the prosecution of this subject, attempt to show which of the opinions has the strongest claims to public confidence." (P. 76.)

In the opinion of our author, experience has abundantly proved that, if the impregnated uterus be retroverted, and not restored to its natural position, it will go on to augment in size, and at last completely occupy the cavity of the pelvis. The opinion of Dr. DENMAN, that a distended bladder is always the immediate cause of retroversion, is properly objected to. "1st. Because we are certain that it has been suddenly produced by violence, and without the intervention of a suppression of urine. Baudelocque also declares the same thing. 2d. Because Baudelocque demonstrated to his class a slow retroversion of the uterus, which lasted three or four weeks before it was complete: in this case there was no mention of any difficulty in making water." (P. 78.)

"The uterus should in every instance be restored, when practicable, at or very little after the fourth month: if left longer than this, the risk of not succeeding is every day increased; and we are firmly of opinion that nothing can justify a neglect to do so at this time, more especially when it proceeds from the vain hope that nature will relieve herself at the full period of gestation." (P. 80.)

We should recommend the perusal of Mr. BURNS' observations upon the subject of retroversion of the uterus. For whatever purpose the hand is introduced into the vagina, we shall have to contend with the difficulty which will arise from "the violent and involuntary efforts to bear down, to which the woman is excited by the presence of the hand within the vagina. This is decidedly the greatest trouble we meet with in ordinary cases; for we may be foiled in our attempts at reposition, though the emptying of the bladder and rectum should not have been found troublesome. To overcome this opposition, experience has repeatedly taught us the efficacy of bleeding to, or near to, fainting." (P. 83.)

*Of the Obliquities of the Uterus.*—The anterior obliquity of the uterus is a source of great inconvenience to the woman, even before labour. After the seventh month, the fundus is so precipitated, and in advance, as to destroy the common centre of gravity, and the woman is obliged by exertion to make herself a new one, when she either walks or stands. In our own practice, we have been frequently required to remedy this inconvenience, and to suggest an effectual bandage has been considered an easy duty. We have tried various bandages, however, and none with satisfaction to ourselves, nor to the effectual relief of the patient. The contrivance of Dr. Dewees appears likely to succeed. He employs "a pair of drawers, with a waistcoat attached to it which will lace behind. The waistcoat need not reach but a little way above the umbilicus, but must be maintained in its proper situation by a support from above, by a pair of properly adjusted suspenders. This dress should be put on in the morning before the woman rises upon her feet; and, when it is applied, the fundus should be raised by the patient's own hands, locked together, being placed under it, and lifted as it were upwards, while the nurse, or a friend, should lace the back part of the waistcoat as tight as will give support to the uterus, when left to itself to press against it." (P. 88.)

"In cases of extreme obliquity, it is oftentimes difficult to reach the os uteri by the ordinary mode of examination: when this happens, the pendulous belly should be raised and supported by an assistant, with a view to depress the os uteri: should this not succeed, and should the pains be brisk, the head will be found to sink lower and lower in the pelvis, covered by the stretched anterior portion of the uterus. If advantage be not now taken to introduce the hand to restore the os uteri to the proper axis of the pelvis, much suffering must be endured, and much risk incurred, by permitting the head to descend covered by the uterus.

"Whenever it is found that the os uteri cannot be reached by a well-directed search in the ordinary way, we must introduce the hand, well lubricated, so that its palm may be next to the distended uterus; a finger should then be made to reach up to the neighbourhood of the projection of the sacrum, where, on some one portion of the uterine globe, the os uteri will be detected. When discovered, we should hook it upon the point of the finger, (provided it is either dilated or easily dilatable,) and draw it towards the centre of the inferior strait: when it has followed so far, the hand may be gently withdrawn, (but not the finger from the os uteri,) and the uterus detained there until the proper direction of the forces and the axis of the uterus are made to correspond. By this simple proceeding, much time and suffering are saved; and, in some instances, we are well persuaded that much risk is prevented. Baudelocque has most satisfactorily illustrated the advantage of judicious interference, and the neglect of it, by the recital of two opposite cases, to which we would refer the reader, with much advantage to himself. (P. 89, 90.)

We can conceive a case of such obliquity of the uterus, that it may be necessary for us to endeavour to place that organ in its proper position. In the course, however, of a very considerable number of labours, which we have had under our management, no such instance has occurred. We most decidedly object to, and we strongly deprecate, the unconditional and indefinite advice given by Dr. Dewees, that, "whenever it is found that the os uteri cannot be reached by a well-directed search in the ordinary way, that we must introduce the hand." Is it meant that we are to introduce the hand, if we fail in finding the os uteri at a *first* and "well-directed" examination?—or are we to wait some time, in the expectation that, as the labour advances, the os uteri will become within reach of the finger? The introduction of the hand into the vagina is, in first labours, and at the commencement of the process, a very painful operation for the patient, and not always of very easy execution to the practitioner. In innumerable instances, where there has existed a slight anterior obliquity of the uterus, we have not been able with the finger to reach the os uteri. We have not felt ourselves called upon, however, immediately to introduce the hand. We have waited for some time, and have always found the os uteri brought within our reach by the natural efforts. When we recommend the propriety of delay in such instances, it is, of course, upon the supposition that there are no urgent symptoms which demand our active interference.

*Upon the Signs which usually accompany Pregnancy*, we must be brief. This is a fertile theme for discussion, upon which there are scarcely two opinions in unison. Dr. Dewees adds his testimony to the fact, which we consider to be now well established,—namely, that women may menstruate during pregnancy. In one extraordinary case which fell under his observation, the menses, which had been previously suppressed, were produced by pregnancy. "The areolæ, which are sometimes formed round the nipple, must be considered as equivocal in any but a first pregnancy: in this case, did areolæ form, we should place great dependence upon them, having so far never been deceived." (P. 98.) The appearance of milk in the breasts is, in the opinion of the vulgar, an infallible sign of pregnancy. We know that it is not so. A distressing case is related by Dr. Dewees, in which a young physician positively determined a lady was pregnant, who had been two years separated from her husband, because the mammæ contained milk. After an examination, Dr. D. pronounced her not pregnant; and the result proved that he was correct. The patient died, "eight months after, of phthisis pulmonalis, in which the obstruction of the catamenia is not an unfrequent occurrence."

Need we add, after the recital of such a striking example, that it behoves every man, who has his own reputation at heart, and who is not insensible to the undeserved misery he may inflict upon his patient, to hesitate before he ventures to give a certain opinion upon so uncertain a subject. In brief, candour demands the confession that we at present know no symptom, or assemblage of symptoms, which *positively* indicates pregnancy. Every symptom usually considered as an evidence of pregnancy, may exist when the woman is not pregnant; and she may be pregnant, without the occurrence of a single symptom which would lead to a suspicion of her state. We speak of possibilities, and are aware that such cases do not very commonly occur. There is even some difference of opinion as to the period of utero-gestation, when we shall be able, by examination *per vaginam*, to determine positively the existence of pregnancy. Upon this subject our author does not touch.

*Vomiting during pregnancy* has frequently resisted every remedy in our own practice. Dr. Dewees rarely perseveres in the use of the alkaline remedies, when he finds that considerable doses will scarcely have a temporary effect. When this is the case, he has recourse to acids themselves for the relief of this most distressing state of the stomach. He has also repeatedly found benefit from spirits of turpentine, exhibited in doses of twenty drops three or four times a-day.

In cases of obstinate *Fluor Albus*, warm water is advised as a wash.

"We are aware we differ, in recommending, as a common wash warm water, from almost every other practitioner; but we feel in this we are recommending the better plan: it is one we have adopted for the last thirty years, and are abundantly convinced of its superiority over the other." (P. 117.)

On the subject of *Pruritus* of the pudendum, which we have frequently known to be the source of very distressing torment for a considerable time, we receive every novel suggestion as to treatment with gratitude. Dr. Dewees recommends a strong solution of borax in water, which is to be employed as a wash and an injection four or five times a-day. In one very severe case, relief was obtained in twenty-four hours. "We were led," says Dr. D. "to the employment of the borax, from the analogy which the thrush in children furnished us with; and its uniform success since has led us to believe it to be nearly certain in this complaint," (pruritus.) If the case is very obstinate, local bleeding and purging are considered necessary. Dr. D. has only twice had an opportunity of examining the parts during this affection, and in both these cases they were covered with an apthous efflorescence.

*Prolapsus Uteri.*—The candour of our author deserves our acknowledgment, while at the same time it conveys useful instruction.

"Notwithstanding the diagnostics of this complaint are so strongly and decidedly marked, yet they are not sufficiently so to warrant us in taking this for granted: we should never, but from a careful examination, pronounce this complaint positively present, lest we may be in error, as once happened to ourselves. We were consulted by a lady, who had present almost every symptom recorded above: we, without hesitation, pronounced her disease to be a prolapsus of the uterus, and, without further examination, had a pessary made for its support; but, to our sad mortification, when we were about to apply it, a careful examination proved that no such condition existed, and that all the unpleasant symptoms had arisen from a thickening of the neck of the bladder." (P. 127.)

Upon the subject of *Menstrual Derangement*, we perfectly accord with Dr. Dewees. Humanity would shudder, if it were possible to collect a list of even half the cases in which the lives of females are sacrificed, by the temerity of quacks, the obstinacy of nurses, and the folly of mothers, in endeavouring "to bring down the courses." "Our rule," says Dr. D. "on this point constantly is, and hitherto we have seen no cause to weaken our reliance on it, never to interfere, unless there be some evidence that the health is suffering by the absence of this discharge," (the menses.)

Fluor albus accompanying menstrual suppression is sufficient to call for our aid.

Dr. Dewees does not consider any case "an immoderate flow of the menses," unless it affects the health. Menstrual excess is a relative term: the quantity discharged may be excessive to one patient, and scanty to another. We are quite convinced that nine-tenths of the cases of immoderate menstruation, particularly those which occur in the higher circles, where something medical must be doing, are imaginary. During the long and active career of Dr. Dewees, he has met with but one case to which he would apply this term.

By some authors, the sugar of lead is deprecated as a dangerous remedy in *menorrhagia*, or uterine hemorrhage, properly so called. Dr. Dewees has used it freely, without inconvenience. We have administered it ourselves very frequently, and look upon it as a most valuable and perfectly safe remedy. A grain twice a-day is the dose we have been accustomed to commence with, in combination with a quarter of a grain of opium. It will frequently be necessary to increase the quantity considerably, but we should do it gradually.

*Dysmenorrhœa* is very common in America. Camphor, in ten-grain doses, is recommended, which may be repeated every hour, if necessary. We have offered our opinion above upon the propriety of beginning with these doses. The volatile tincture of guaiacum is considered a valuable remedy.

"When speaking of the tact that is acquired in the administration of certain medicines in certain diseases, we had particular reference to the employment of the tincture of guaiacum as an emmenagogue. We have, for nearly five-and-thirty years, almost daily used this medicine in suppressed catamenia, and more especially in those of long standing, without its having failed in any case proper for its use:* more cannot be said of any remedy whatever.

"We say this in the most perfect good faith, as we have learned that some of our brother practitioners have not been equally successful with it; but we think we can readily account for their failure: 1st, from their not placing the system in a proper situation for its use; and, 2d, by not properly persevering in it. Neglecting these important points, it can readily be imagined it may not succeed; as we deem an attention to them essential to its success, more especially in those cases where many months of interruption have existed. We think one of its superiorities consists in its certainty in cases of very long standing; and we could readily furnish from our note-book a number of instances where it succeeded, after a lapse of from nine months to nearly three years.

"The mode of using it is, a tea-spoonful every morning, noon, and evening, in a wine-glassful of sweetened milk, or (where not forbidden by some peculiarity or circumstance,) as much white wine,—as sherry, Teneriffe, or Madeira. The dose must be gradually increased in those cases where a perseverance beyond four or five weeks becomes necessary. Should this medicine disturb the bowels too much, a few drops of laudanum must be added to each dose; but if, on the contrary, they should not be sufficiently opened, the addition of a little of the resin of jalap, or powdered rhubarb, will be an improvement.

"As the tincture we employ is different from the tincture of the shops, we think it right to subjoin our formula:

℞ Pulv. G. Guaiac. opt. ℥iv.

Carbon. Sod. vel Potas. ʒjss.

Pulv. Piment. ʒj.

Alcohol. dilut. ℔j. Digest for a few days.

"The volatile spirit of sal ammoniac to be added pro re nata, in the proportion of a drachm or two to every four ounces of tincture; or less or more, agreeably to the state of the system." (P. 143—145.)

*On the Term of Utero-gestation.*—As we have no certain mark to date conception from, we cannot ascertain, with precision, the period the fœtus resides in utero.

"But opportunities occasionally occur, where the utmost accuracy must prevail: one of this kind presented itself to our notice many years ago. The husband of a lady, who was obliged to absent himself many months, in consequence of the embarrassment of his affairs, returned, however, one night clandestinely, and his visit was only known to his wife, her mother, and ourselves. The consequence of this visit was the impregnation of his wife; and she was delivered of a healthy child in nine months and thirteen days after this nocturnal visit. The lady was within a week of her menstrual period, which was not interrupted,

* "By a proper case, we mean where the suppression is an idiopathic disease, and not one where the uterus has its functions interrupted by other diseases, or pregnancy: for we confess, in the latter, we have in two or three instances been imposed upon, notwithstanding all our caution, and where we dared not suppose this condition to exist; but by these few cases, we learnt, so far as they go, it would not produce abortion."

and which led her to hope she had suffered nothing from her intercourse; but the interruption of the succeeding period gave rise to the suspicion she was not safe, and which was afterwards realised by the birth of a child." (P. 165.)

This case deserves attention on three accounts:—1st. A woman in perfect health, and pregnant with a healthy child, may exceed the period of nine months by several days. 2d. She may be impregnated just before her menstrual period, and not have that interrupted; and therefore, 3d, a check is not immediately given to the catamenial flow, by an ovum becoming impregnated. These are very important points. In judicial inquiries under particular circumstances, the most lamentable consequences might arise from the practitioner not being aware of them.

*Of Labour.*—Certain symptoms usually precede the occurrence of labour, such as rigors and a train of nervous symptoms. Rigors are very common: they are not indicative of any febrile movement, and rarely require attention. The common practice of giving the patient hot drinks and stimulating liquors, for the purpose of relieving this symptom, which is not indeed attended by any sensation of cold, is decidedly improper. We must refer to the work itself for the detail of the various constitutional symptoms which accompany an ordinary and healthy labour. The uterine system undergoes particular changes, as, "*a*, the subsiding of the abdominal tumor; *b*, the secretion of mucus; *c*, the dilatation of the os uteri; *d*, the alternate contractions of the uterus."

"It has ever been justly considered a favourable circumstance when the sinking of the uterus takes place, as it would seem to declare two important facts connected with delivery:—1st, a healthy condition of the uterus itself; and, 2d, a healthy conformation of the pelvis." (P. 171.)

A secretion of mucus almost always takes place before other symptoms show labour to be at hand. It is a welcome harbinger to the experienced practitioner. Without this discharge, even if severe pains have occurred, we rarely find much progress made, or a favourable state of relaxation and dilatability of the parts. This mucous discharge is very frequently tinged with blood. "Dr. Denman calls it an increased secretion of the fluid natural to the parts; but to this it does not appear to have the least resemblance, and, even if it be furnished by the same vessels, it must be by an altered action of them."

The following passage should be deeply engraven upon the mind of every accoucheur. Its precepts are too frequently neglected, from the impatient restlessness of the practitioner, who, by his officious meddling, procrastinates the process he is so unwarrantably anxious to hasten.

"The writers on Midwifery have but too constantly limited the use of this discharge to a mere lubricant; and they carefully caution against too frequent touching, because, say they, it removes this substance from the vagina, and thus gives rise to more friction between the child's head and the soft parts of the mother. Now, were this the only evil to be apprehended by incautious or unnecessary touching, it could be easily remedied by any mild unctuous substance; but, as we have observed, it is but too well known, if not acknowledged, that this by no means answers the purpose for which we believe this discharge was instituted.

"By frequent and incautious touching, the glands furnishing this discharge are over-stimulated, or become inflamed: the secretion immediately ceases, the parts become tender and swoln,—especially the mouth of the uterus, if not fully dilated; the pains will become less frequent and less protrusive; the woman becomes restless, and enjoys no calm in the intervals of the pains; fever is excited; head-ache, thirst, and a hot skin follow: in a word, a new condition of the system arises, and almost supersedes the business of labour. It would be in vain, under such circumstances, to offer a substitute for the lost mucous secretion, by presenting to the parts any unctuous or mucilaginous substance whatever: it can only be recalled by rest and free blood-letting. To the last we must have immediate recourse, if we wish to subdue the unnecessarily provoked inflammation, and restore the uterus to the enjoyment of its suspended powers. In many cases like those just mentioned, we have seen this remedy act with the certainty and promptitude of a charm.

"The disturbance excited throughout the system when the vaginal surface becomes inflamed, distinctly shows us the important rule the mucous secretion performs in the economy of labour: it demonstrates to us that it is instituted for a much higher purpose than merely to lubricate the parts, that they may transmit the child with more facility;—it shows us clearly that its formation is, in some way or other, connected with the dilatation of the os uteri, and the relaxation of the perinæum; —let us beware, then, how we interrupt its formation by rude and uncalled-for handling." (P. 172, 173.)

[To be continued.]

## Figures and Tables

**Figure f1:**